# The current state of forensic imaging – recommended radiological tools and international guidelines

**DOI:** 10.1007/s00414-025-03465-7

**Published:** 2025-03-25

**Authors:** Fabrice Dedouit, Mathilde Ducloyer, Jamie Elifritz, Natalie L. Adolphi, Grace Wong Yi-Li, Summer Decker, Jonathan Ford, Yanko Kolev, Michael Thali

**Affiliations:** 1 Department of Forensic Pathology, Bâtiment Raymonde Fournet, Place du Dr Baylac, Hôpital Purpan, Toulouse, 31700 France; 2 Department of Forensic Pathology, University Hospital, Nantes University, Bd Jean Monnet, Nantes, F-44000 France; 3 Forensic Radiology Group, Anderson, SC USA; 4 Office of the Medical Investigator, University of New Mexico, Albuquerque, NM 87131 USA; 5 Department of Radiology, Penang General Hospital, Jalan Residensi, Georgetown, Penang 10450 Malaysia; 6 Departments of Radiology and Pathology, University of Southern California Keck School of Medicine, 1450 San Pablo Street, Suite 3500, Los Angeles, CA 90033 USA; 7 Department of General Medicine, Forensic Medicine and Deontology, Medical University-Pleven, 1 St Kliment Ohridski Str, Pleven, 5800 Bulgaria; 8 University Zurich, Virtopsy Group, Switzerland

**Keywords:** Forensic imaging, Forensic science, Postmortem computed tomography, Recommendations

## Abstract

The last few decades have seen the emergence of forensic imaging, both clinical and post-mortem. Year after year, the scientific community has refined the radiological tools that can be used for post-mortem and clinical forensic purposes. As a result, scientific societies have published recommendations that are essential for the daily work of forensic imaging. This third part of the review of the current state of forensic imaging describes these recommended radiological tools and also presents an overview of the various international guidelines dealing with post mortem imaging that can be found in the literature or that have been written by scientific societies.

## Introduction

**Fig. 1:**
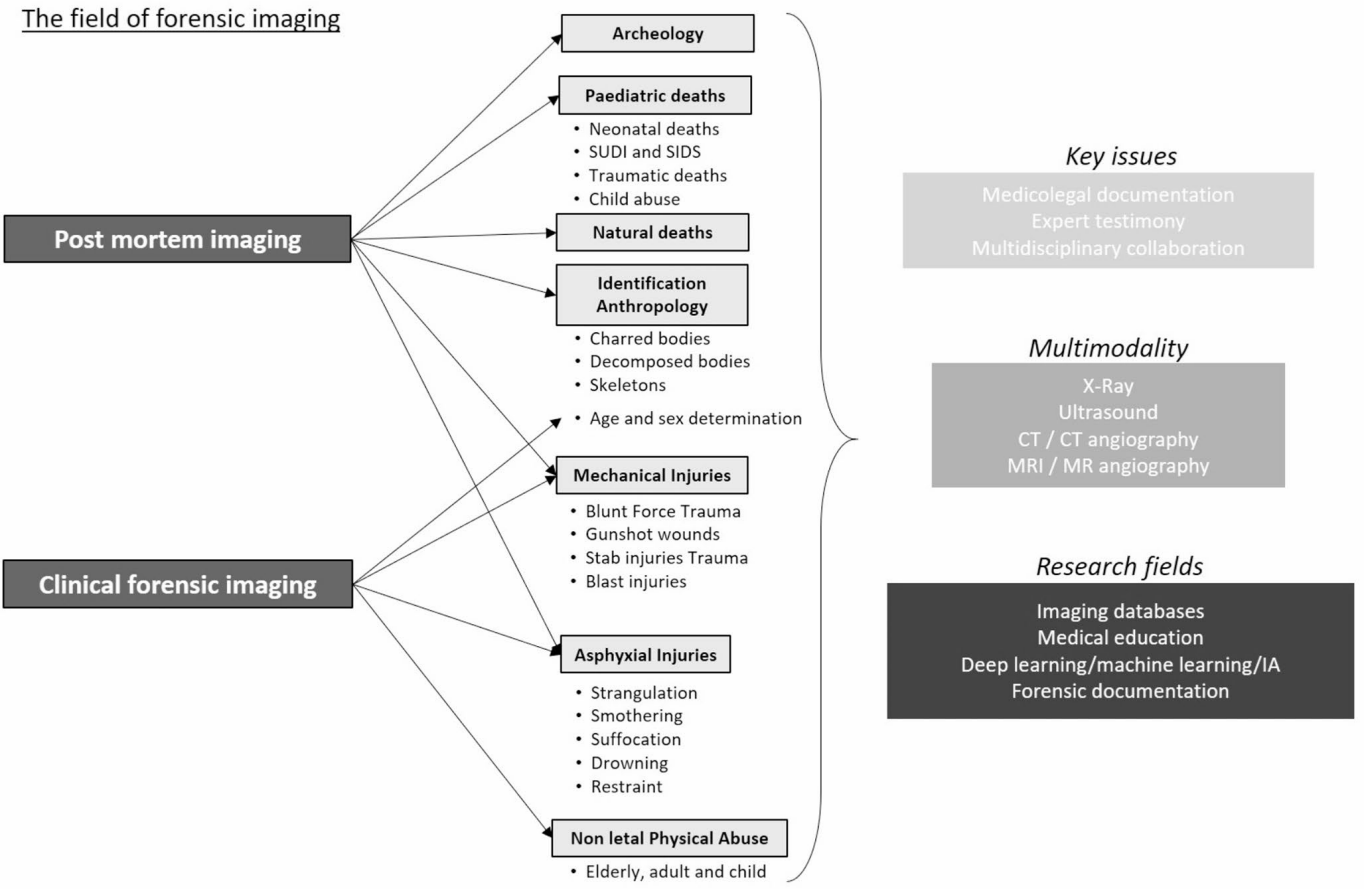
Illustration of the main indications and benefits of post-mortem imaging in the most common forensic situations

Forensic imaging includes all imaging techniques that can be related to forensic science. Of course, classical radiological tools can be used in forensic radiology: radiography (computed radiography (CR) and digital radiography (DR)), multislice or multidetector computed tomography (MSCT or MDCT), and magnetic resonance imaging (MRI) [1–4]. More specialised tools such as micro-CT, micro-MRI and MR spectroscopy can also be used [5]. New techniques now allow contrast agents to be injected into cadavers, producing high quality images [6]. This multimodality is illustrated in Fig. 1. The forensic pathologist or radiologist performing forensic imaging must be aware of the advantages and limitations of these radiological tools. Interestingly, in the jungle or void of possible recommendations in post-mortem imaging, some scientific societies have written recommendations on the indications of PMCT in a thanatological context. These recommendations are helpful for any radiologist or forensic pathologist who wants to implement post-mortem imaging in the workflow of a medico-legal cadaver in the forensic pathway, from imaging to external examination and autopsy.

The aim of this third part of the review of the current state of forensic imaging is to describe the different imaging modalities and their application in post-mortem imaging, as well as the different guidelines published by the scientific societies of forensic imaging.

## Recommended radiological tools for post mortem and clinical uses

### X-ray

Conventional x-ray imaging was the first radiological imaging technique used in forensic medicine, dating back to 1896 [7]. Conventional x-ray is still widely used in death investigation and may be the primary (or only) radiological imaging modality available in some jurisdictions. Conventional x-ray is a relatively simple and low-cost technology, with utility for applications such as detecting fractures or foreign bodies, and for identifying a deceased subject through comparison of post-mortem and ante-mortem dental x-rays [2].

Conventional x-ray imaging uses high energy photons produced by an x-ray source to cast shadows onto an image receptor, revealing internal structures in the subject, who is placed between the source and receptor. Image contrast is based on the electron density of the subject’s tissues and other objects that may be present. Heavier elements, such as calcium and phosphorus in bones, have higher electron densities and therefore are more attenuating of the incident x-rays, compared to normal soft tissues [8]. Image receptor types include film, CR (computed radiography) cassettes, and DR (digital radiography). Because images are formed by shadow-casting, a computer is not strictly necessary to produce a basic image, and the first type of image receptor was simply radiosensitive film. Digital processing, using CR or DR has many advantages, including avoiding consumables such as film and the wet chemicals required to process it [9]. Further, modern algorithms applied to digital x-ray image data can be employed to enhance image quality. DR is especially convenient, because the image can be processed digitally while the subject remains positioned on the image receptor, making it much easier to correct poor positioning or the incorrect choice of technique.

The types of x-ray units used in forensic imaging include fixed systems (“x-ray rooms”), portables, whole body x-ray scanners (see for example Morele et al.), C-arms, cabinet x-ray units, and handheld dental x-ray units (see for example Pittayapat et al.) [10, 11]. Novel battery-powered handheld x-ray units, designed for imaging the extremities and body, are under development and may someday prove useful in forensic field work, such as anthropology and mass disaster situations. Fluoroscopy is a type of x-ray imaging involving a continuous display on a monitor, essentially an x-ray video. Fluoroscopy can be used to capture still images and/or cine. In the forensic setting, fluoroscopy may be used for applications such as searching for foreign bodies or guiding needles for biopsy sampling or contrast administration [2].

The resulting images from x-ray are inherently two-dimensional, resulting in overlap of the internal structures and a lack of depth perception. Grayscale values range from white (high density) to black (air or gas). Because the grayscale at any point in an x-ray image depends on the sum of the attenuation properties of all tissue types and materials and their respective thicknesses, the image grayscale cannot be calibrated to indicate which materials are present. This ambiguity in the grayscale values is referred to as “summation artifact” [12]. Another artifact present in x-ray imaging is magnification. Anatomic structures in direct contact with the image receptor are minimally magnified, but as the distance between the anatomy and the image receptor increases, the magnification increases. Therefore, the magnification within a single 2D image is not consistent (for example, anterior ribs are magnified relative to posterior ribs), and sizes measured from conventional x-ray, such as the diameter of a bullet, are not accurate without a correction for the distance to the image receptor [4]. However, one advantage of conventional x-ray over computed tomography is that conventional x-ray imaging is not subject to metal artifact, which is present in CT and MRI, as discussed below.

### Post mortem Computed Tomography (PMCT) and Post mortem Computed Tomography angiography (PMCTA)

X-ray CT is finding increasing use in death investigation and has become the primary radiological imaging modality used in a number of jurisdictions around the world. PMCT can be considered the current state-of-the art in forensic imaging [1]. PMCT is used to detect fractures, the precise location of foreign bodies, as well as many other injuries and pathologic conditions, made possible by the 3D nature of the resulting image data sets [2]. PMCT is also used for image-guided biopsy in minimally invasive autopsy and for PMCTA [13–15].

Like conventional x-ray imaging, CT imaging is also based on high energy x-rays and their differential attenuation according to the electron densities present in the subject. It differs from conventional x-ray in that the x-ray source and image receptors rotate rapidly around the subject (within a torus-shaped housing known as the gantry), while the subject moves (on a cantilevered table) through the centre of the gantry. Cross-sectional image data is not immediately available and must be computed (“reconstructed”) from the raw spiral data captured by the rotating x-ray detectors. Multiple rows of x-ray detectors (from 4 detectors rows up to 128 or more) improve the efficiency of imaging, increasing the acquisition speed and resulting image resolution [8]. Newer CT scanners have sufficient scan length (i.e., maximum distance the table travels), up to 2 m, to enable whole body scanning of most adult subjects in a single scan [16].

Types of CT scanners include stationary, portable, micro-focus, and photon-counting detector CT scanners. Traditionally, CT scanners have a stationary gantry, built into a dedicated CT room with radiation shielding. Recently, portable units have become available, where both the gantry and table can be moved (on wheels) and these units may be used (with precautions) in an unshielded environment [17]. Generally, the portable units are capable of imaging specific anatomic regions (such as the head) but are not designed for whole body scanning. Micro-focus CT scanner (“micro-CT”) are intended for high resolution imaging of small samples, which are enclosed in a cabinet. In a micro-CT scanner, the x-ray source and detectors are stationary and the sample is rotated on a stage. In the post-mortem imaging context, micro-CT can be used to produce highly-detailed images of small specimens, such as teeth, bone fragments, foreign bodies, insects, or a human foetus [5, 18]. Photon-counting detectors (PCD) are a relatively recent technological advance over the traditional, energy-integrating detectors (EID) found in most CT scanners. PCD detectors enable higher spatial resolution, improved contrast-to-noise, and energy resolution not available using conventional EID detectors [19]. These advantageous features of PCD are likely to find application in post-mortem imaging in the near future.

Some CT scanner technical specifications of interest to medicolegal death investigators are related to the size of the subject who can be imaged. These include the maximum scan length, gantry bore diameter, and maximum reconstructed image diameter (field-of-view). A large bore diameter ensures that large subjects or bodies that are charred or bloated can pass through the gantry to be imaged. A large image field-of-view ensures that the extremities (and not just the thorax) are included in the reconstructed images [20].

The data resulting from CT is fundamentally digital and three-dimensional and can be reconstructed or reformatted to optimize image quality for different tissue types and to display different viewing planes. Whole body CT data is typically reconstructed using both soft tissue-optimized and bone-optimized image reconstruction settings, and other specific image processing settings may be used for the brain, the lungs, or other specific tissues [21, 22]. CT image reconstruction avoids the magnification and summation artifacts seen in conventional x-ray, resulting in an image appearance that is qualitatively clearer and more realistic-looking, similar to the appearance of gross pathology sections (but without color). The 3dimension enables depth perception and precise localization of injuries or foreign bodies, absent in conventional x-ray.

Quantitatively, the absence of magnification artifact and summation/overlap artifact enables both the geometry and grayscale (density) of CT images to be calibrated to a high level of accuracy. Distances and sizes can be measured from CT with an accuracy only limited by the pixel dimensions and slice thickness of the cross-sectional images. The image voxels in CT are typically of order 0.5 to 1 mm in the axial plane and 1 to 10 mm in slice thickness. The grayscale (i.e., the CT number) is calibrated in Hounsfield Units (HU), with − 1000 HU denoting air or gas, 0 HU denoting water, up to + 3000 HU, which exceeds the density of bone. This calibration enables specific tissues, fluids, and pathologic conditions to be identified based on the CT number [23]. Dual energy CT acquisition (which involves acquiring images of the same subject at two different x-ray energies) enables even more precise material identification [24].

CT is more powerful than x-ray for identifying subjects by radiological comparison, due to the possibility of displaying CT image data in whichever plane best matches the available antemortem medical images [25, 26]. For example, curved multi-planar reformatting enables the creation of CT-derived (synthetic) orthopantomograms for comparison to ante-mortem dental x-rays [27, 28]. The 3D nature of the data also enables forensic anthropologists to perform osteometric analyses from image data, with an accuracy comparable to that of direct physical measurements of dry bones, for the purposes of age, sex, or ancestry estimation [29]. Finally, the 3D data enables a variety of useful visualizations to be produced, including 3D surface rendering, cinematic rendering, and 3D printing, techniques discussed in greater detail below [27].

While CT avoids common artifacts of conventional radiography, there are other artifacts that arise which are specific to CT image reconstruction, CT hardware, and subject characteristics [30]. The most commonly-encountered artifacts in the forensic imaging setting are out of field artifact and streak artifacts. Out of field artifact occurs when part of the subject anatomy is outside of the reconstructed image field of view, which may occur when the body is large or cannot be positioned properly due to charring or mummification. Streak artifact may appear as dark streaks between two dense objects (for example, in the posterior fossa) or alternating bright and dark streaks surrounding a single high-density object, such as a bullet or metallic implant. While streak artifact caused by metal may obscure the soft tissue immediately surrounding the metal, metal artifact is, to some extent, helpful for quickly locating metal fragments and differentiating them from bone fragments. Metal artifact reduction algorithms are an available add-on for improving the image quality of tissue in the presence of metal [31].

PMCT is often performed without contrast enhancement. However, PMCT angiography (PMCTA) is a well-established method used at a number of forensic centres around the world. To perform angiography, a contrast medium (a fluid that highly attenuates x-rays) is delivered to the vasculature to dramatically increase the image contrast between vessels and surrounding soft tissues. Angiography enables a significantly more detailed visualization and evaluation of cardiovascular injuries or pathology, such as blockages, leaking, lacerations, or rupture. In the post-mortem setting (where subjects lack spontaneous circulation), the contrast medium may be delivered locally using a syringe, or it may be circulated throughout the body using a pump [32, 33]. In the case of foetal imaging by micro-CT, contrast is delivered via diffusion, by immersing the subject in the contrast medium [18].

### Magnetic Resonance Imaging (MRI)

While CT produces the highest-quality images of bone, MRI is known for its superior soft tissue contrast, making it the preferred modality for imaging complex soft tissue structures, notably the heart and brain [34–36]. Although less commonly-used than PMCT, postmortem MRI (PMMR) is finding increasing application to death investigation and is currently a topic of considerable research interest in forensic radiology. PMMR is particularly well-suited for imaging paediatric subjects, for whom the CT image contrast is generally poorer relative to the contrast achieved in adult subjects [37, 38].

MRI uses magnetic fields, both static and oscillating, to produce detectable signals from hydrogen atoms throughout the body. Hydrogen nuclei are weakly magnetic, and the collective magnetism from large numbers of hydrogen nuclei (mostly in water molecules in soft tissues) is the source of the MRI signal. The MRI magnet is toroidal, like a CT gantry, but operates on a completely different physical principle. Inside the toroidal shell is a superconducting solenoid that produces a strong magnetic field, typically exceeding 1 Tesla [39]. The most common field strengths for (adult) whole body imaging are 1.5 Tesla and 3 Tesla, although 7 Tesla scanners are now available in some hospitals. Higher resolution post-mortem imaging of ex vivo tissue or foetuses may be performed in small bore magnets with even higher field strengths [40]. In addition to the main magnet, radiofrequency (RF) detectors (“coils”) are placed in proximity to the anatomy to be imaged for the purpose of detecting the hydrogen signals. Other coils are embedded in the magnet bore and are used to deliver pulses of RF energy to excite the hydrogen nuclei and to create spatially-varying magnetic fields to localize the signals in space (the latter being essential for forming an image). Like CT, the raw data from MR (in this case, oscillating voltages) must be processed using a computer to form an anatomic image [39]. Notably, MRI does not employ ionizing (high energy) radiation, and is, in the radiation safety sense, safe for both subjects and operators. For forensic examination of living subjects, particularly children, MRI is often the preferred modality for this reason, in addition to its sensitivity to soft tissue injury. However, we should note that the high magnetic field can exert a very high force on ferrous metallic objects, turning them into high-speed projectiles. Therefore, MRI is not without safety considerations [41]. In the post-mortem imaging setting, subjects with unknown medical history or suspected presence of foreign bodies should receive a PMCT or x-ray examination prior to PMMR, to screen for non-MR safe metallic materials on or in the body [3].

While X-ray and CT contrast are based on material density (which is intuitively easy to understand), MRI contrast is based on the time-dependence of the oscillating voltage signals from each tissue, which is a complex subject. In fact, manipulating the timing of the pulses of RF energy in an MRI acquisition sequence can be used to manipulate the tissue contrast quite significantly [42]. As an example, in a brain image resulting from one type of MR acquisition sequence, gray matter will appear brighter than white matter, but using a different MR acquisition sequence, the contrast will be reversed. The two most basic types of MRI contrast are referred to as “T1-weighted” and “T2-weighted,” referencing the relaxation times T1 and T2 which are unique for each tissue type. However, many other endogenous contrast mechanisms, such as inversion recovery, diffusion, and magnetic susceptibility-weighting, can be utilized to produce a wide variety of MRI contrasts [39]. Like PMCT, PMMR is most often performed without (exogenous) contrast. Nonetheless, post-mortem MR angiography is a feasible option for combining the soft tissue contrast of MRI with the additional information provided by angiography and technical developments in post mortem MR angiography are a topic of continuing research [43–46].

Due to the exquisite sensitivity of MRI to variations in soft tissue, including tissue temperature, PMMR is sensitive to normal post-mortem changes, and standard acquisition sequences may need to be adjusted to maintain image quality for post-mortem subjects [47]. Further, MR is subject to its own unique image artifacts. Clinically, the most common MR artifacts are caused by subject motion, including blood flow and peristalsis, but motion-related artifacts are almost entirely avoided in PMMR [3, 48]. The most likely MRI artifacts in the post-mortem setting are aliasing (or wrap-around) artifact, which occurs when subject anatomy is outside of the imaging field of view, and magnetic inhomogeneity artifact, which occurs due to abrupt spatial variations in magnetic susceptibility, such as air-tissue interfaces or the interface between tissue and foreign bodies. If the foreign body is metallic, particularly if it is a ferrous metal, the resulting image artifact may be severe and is another reason to screen for metallic objects by CT or x-ray prior to PMMR.

### Ultrasound (US)

Ultrasound (US) generally yields poorer image detail than is achievable from CT or MRI, but it is nonetheless useful for a number of specific applications and is widely-used clinically for reasons of speed, portability, safety, and low cost. However, to date, US is only rarely used in the forensic setting [49, 50]. Forensic applications include post-mortem perinatal imaging, image-guidance of needle placement and age estimation in living paediatric subjects [51–54]. US employs high-frequency sound waves to interrogate underlying anatomic structures. Like MRI, US does not involve ionizing radiation, and is therefore safe for both living subjects and technologists. US imaging systems are small and easily portable. Sound waves are transmitted from a hand-held transducer placed in direct contact with the subject’s skin. Sound waves travel from the transducer through the body and are reflected most efficiently from boundaries between dissimilar tissues (e.g., fluid/bone, air/tissue). Reflected sound waves are detected at the transducer and converted to electrical signals. The timing, strength, and location of the signals arriving at the transducer are used to compute the position and depth of the underlying anatomic structures, resulting in an image. Typical US systems produce 2D images of a cross-section through the anatomy of interest. 3D and 4D US imaging is possible but less common. Doppler US (used to measure flow in the clinical setting) is not relevant to post-mortem imaging [8].

### Recommendations edited by national/international societies

#### Current PMCT guidelines

Few guidelines exist for Postmortem computed tomography (PMCT) and the approach to this topic is varied. For instance, some nations/organizations approach guidelines from a broad perspective of forensic imaging, and some are more specific to PMCT. Many are organized by distinct PMCT applications, and one is organized by specific individual findings. The following references were deemed relevant to the scope of recommendations edited by national/international societies [55–63]:

1. Forensic Imaging Working Group of the Medical Section of the Swiss Society of Forensic Medicine (SGRM)
2. Forensic Imaging Working Group of the German Society for Forensic Medicine (DGRM)
3. Postmortem Imaging Interpretation Guidelines 2015 In Japan
4. European Society of Paediatric Radiology (ESPR)
5. International Association of Forensic Radiographers (IAFR)
6. The Royal College of Pathologists
7. Italian Society of Medical and Interventional Radiology
8. Health Professional Counsel of South Africa
9. Rutty’s Rules

Table 1 summarizes these recommendations.

**Table 1:** Summary of the recommendations dealing with PMCT recommendations of the included national and international societies

Organization	Published date	PMCT applications	PMCT limitations	PMCT technical recommendations	Education and training recommendations	Other high yield topics
Swiss	2014	*Not specifically listed *Note that PMCT is a routine practice on most cases	*Not specifically listed	General recommendations	“Experts”	Radiation protection, preparation and transportation of the body, preparation of the body, Data archiving
German working group	2015	*Written specifically for PMCT	*Non-displaced rib fractures *Foreign bodies with attenuation similar to human tissue *Reconstruction of wound channels *Type of gas *Coronary thrombosis *Myocardial infarction	General recommendations	General recommendations	Radiation protection, infrastructure, preparation and transportation of the body, data archiving, personnel, teleradiology, demonstrative aids in court
		PMCT as a rule: *Homicide *Radiopaque foreign bodies *Child abuse/infanticide *Unexpected deaths in young children (up to 6 years) *Air/gas embolism				
		PMCT case by case basis: *Accidental death *Suspected treatment errors Unexpected deaths up to age 17 years *Highly altered corpses *Unidentified corpses / Radiologic identification *Mass disaster *Practitioner self-protection	*Pulmonary embolism *Sepsis *Intoxication			
Japan	2015	*Traumatic death *Drowning (caveats) *Carbonization (caveats) *Asphyxia(caveats) *Intoxication (caveats) *Hypothermic deaths (caveats) *Gas embolism (caveats) *Chronic physical abuse (caveats) *Natural (caveats)	*Myocardial infarction (caveats) *Pulmonary embolism (caveats)	N/A	N/A	Legal requirements for forensic imaging, article is specific to death occurring outside medical institutions, MRI
ESPR and ISFRI guidelines	2019	*Written specifically for PMCT *Applications not specifically listed *Case-by-case in routine practice	N/A	Detailed acquisition protocol for children	Trained radiographer Qualified radiologist	Forensic imaging in children, international survey, routine practice, standardization for research
IAFR Guidelines	2020	*Not specific to PMCT *Sudden unexpected adult death *Sudden unexpected infant death *Road traffic accidents *Homicide *Suicide *Overdose *Deaths following medical intervention *Custodial death *Decomposed remains *Mass fatality *Sudden unexplained death in epilepsy	N/A	N/A	Specific for radiographers	Forensic imaging of the living, evidence, protocols, consent, confidentiality, medico-legal, records, involvement of students and assistant practitioners, reports, health and safety, mass fatality, employers
Royal college of pathologists	2021	*Specifically, “cross-sectional imaging”	Cannot be reliably diagnosed using nonenhanced cross-sectional imaging:	N/A	General recommendations	Audit, health and safety, PMCT angiography, image guided biopsy, toxicology
		Mentions: *Major trauma *Overdose *Natural death	*Sepsis (without abscess) *Toxic ad metabolic conditions *Primary inflammatory diseases			
		Recommends: *The decision for invasive autopsy should be made after postmortem imaging and external examination	*Pulmonary embolism *Intestinal ischemia			
SIRM Italian	2021	*Major trauma *Identification *Gunshot injuries *Battered children *Drowning *Asphyxia *Carbonization *Gas embolism	*Soft tissues *Vascular lesions *Does note improved diagnostic power with PMCT angiography	*Protocols for adults and children	“Specialized radiologist”	Historical perspective, reporting, PMCT angiography, PMMRI
HPCSA	2023	*Not specific to PMCT *Investigation of non-fatal injuries *Foreign bodies *Human Identification *Determining Cause of Death for: *Road traffic deaths *Deaths following medical intervention *Homicide *Suicide *Custodial deaths *Decomposed human remains *Mass fatalities *Sudden infant deaths	N/A	N/A		Responsibilities of employer, responsibilities of radiographer, authentication, evidence, health and safety, records, confidentiality

N/A: not applicable

### Forensic Imaging Working Group of the Medical Section of the Swiss Society of Forensic Medicine (SGRM) [62]

This document was prepared by the members of the Forensic Imaging Working Group of the Medical Section of the Swiss Society of Forensic Medicine. The main purpose of these guidelines is to define minimum requirements for quality management in forensic imaging. The target audience is individuals involved in forensic imaging. The topics included in this document include definition of terms, radiation protection, preparation and transport of the body, generalized PMCT scanning requirements, data archiving, mechanics and personnel considerations regarding interpretation of forensic images, advise regarding additional procedures sharing of information, and applicable literature/documents. Specific applications of PMCT are not addressed.

### German Society for Forensic Medicine (DGRM) Guidelines [55]

This guideline was written by the members of the Forensic Imaging Working Group developed by the German Society for Forensic Medicine (DGRM). The main purpose of these guidelines is to address the practice of PMCT in Germany and the target audience applies broadly to the myriads of professionals involved in this practice. The topics included in this document include indications for PMCT examinations, radiation protection, personnel and material expenditures, the current status of PMCT, infrastructure, preparation and transportation of the body, PMCT scan performance, data archiving, advantages and limitations of PMCT, and teleradiology. The DGRM advise the usage of PMCT in death investigations when technical and personnel resources are available. A distinction is made for indications depending on standard and individual case scenarios. According to the DGRM Working Group, as a rule, PMCT is indicated in homicides, cases with radiopaque foreign bodies, child abuse/infanticide, unexpected deaths in children (up to 6 years of age), and cases of potential air embolism. PMCT should be considered on a case-by-case basis in accidental deaths, suspected medical treatment errors, unexpected pediatric and adolescent deaths (up to age 17), highly altered corpses, unidentified remains, and mass disaster fatalities. Additionally, PMCT can aid in cases the clarify in advance whether there is potential risk to the forensic pathologist and their staff. Coronary thrombosis, myocardial infarction, pulmonary embolism, sepsis, and intoxication are emphasized limitations of PMCT (particularly non-contrast “native” PMCT).

### Postmortem imaging interpretation guidelines 2015 in Japan [58]

This guideline was prepared by numerous stakeholders from hospitals, universities, and the center for cause of death investigation and was edited by the Japan Radiological Society and the Study Group of Japan Health and Labor Sciences Research. It was written in 2015 and the purpose is to serve as a standard for physicians and professionals involved in postmortem imaging. Additionally, this document supports the “Promotion of investigation of causes of death” law in Japan which necessitates postmortem imaging as part of a comprehensive analysis of cause of death related to drugs and toxic substances in Japan. A grading system was developed to approach forensic imaging (multiple modalities) in reference to specific postulated clinical questions. The grading scale is rated A-D and ranges from A. Possible to diagnose readily with postmortem imaging (imaging strongly recommended) to D. Difficult to diagnose with postmortem imaging (imaging not recommended). Generalized PMCT applications include traumatic deaths, drowning, charred remains, intoxication, hypothermic deaths, gas embolism, chronic physical abuse, and natural deaths (many with caveats). Evaluation of pulmonary embolism and myocardial infarction are highlighted limitations of PMCT.

### European Society of Paediatric Radiology (ESPR) [63]

The ESPR and ISFRI guidelines were written jointly by the European Society of Paediatric Radiology and the ISFRI in 2019. Their purpose is to provide a standardised imaging protocol for performing post-mortem CT in children, based on responses to an international survey of current practice. These technical guidelines are intended for radiologists and radiographers who wish to adapt their local acquisition protocol or for centres wishing to start this new activity. Several aspects of imaging technique and acquisition (kV, mAs, slice thickness, etc.) have been considered. These recommendations do not specify the indications for paediatric post-mortem imaging or how it should be interpreted, other than that it should be performed by a trained radiologist.

### IAFR Guidelines [60]

The IAFR Guidelines were written by the International Association of Forensic Radiographers is 2020. The aim of the document is to outline best international practice standards for forensic imaging. The target audience is radiographers and their employers. This document defines important terminology, generalizes applications for forensic imaging, broadly addresses forensic imaging protocols, outlines individuals who may request forensic imaging services, and discusses topics ranging from consent required for imaging to confidentiality and other medicolegal considerations. Recommendations for education and training of radiographers, considerations for students and assistant practitioners, and the role that radiographers play in reporting a professional opinion on imaging studies within the scope of their practice are outlined. Other considerations include health and safety, welfare of radiographers, scenarios of suspected physical abuse, hours of service, advice to employers, and mass casualty situations. Specific applications of PMCT are not addressed, however, generalized indications for postmortem imaging include sudden/unexpected adult and infant deaths, road traffic accident deaths, homicides, suicides, overdoses, medical intervention related deaths, custodial deaths, decomposed remains, sudden unexplained deaths in epileptics, and mass fatality events.

### The Royal College of Pathologists Guidelines [61]

This guideline was written jointly by the Royal College of Pathologists and the Royal College of Radiologists to establish high quality and consistent standards regarding the scope and limitations of postmortem imaging as an alternative or adjunct to autopsy to establish the cause of death in adults. The target audience includes those who authorize/request postmortem imaging studies, pathologists who conduct postmortem examinations, and radiologists who interpret postmortem images. This document specifies that it is not applicable to examinations performed when criminal proceedings are involved. This document clarifies terminology, includes multiple imaging modalities (with an emphasis on PMCT), addresses training and qualification standards for radiographers and forensic imaging interpreters, recommends an audit of postmortem imaging services, and acknowledges health and safety concerns in a forensic imaging environment. PMCT is recommended for major trauma, overdose, and natural deaths. The decision for autopsy is advised to occur after postmortem imaging and external examination. PMCT limitations addressed include sepsis (without abscess), toxic and metabolic conditions, primary inflammatory diseases, pulmonary embolism, and intestinal ischemia.

### Society of Medical and Interventional Radiology Guidelines [59]

This guideline was prepared by the Italian Society of Medical and Interventional Radiology in 2021. The key audience is medical examiners and radiologists. The guideline stresses collaboration between the two specialties for PMCT. Key topics include the technical aspects of whole-body imaging in adults and children, PMCT applications, PMCT reporting, organizational processes, who requests PMCT examinations, picture archiving and communication systems (PACS), postprocessing of PMCT examinations, privacy concerns, and economic considerations. Recommended indications for PMCT include identification of unknown remains, gunshot injuries, major trauma, non-accidental trauma of children, drowning, asphyxia, charred remains, and suspected gas embolism.

### Health Professional Counsel of South Africa 2023 [56]

This guideline is written by the Professional Board for Radiography and Clinical Technology of the Health Professions Counsel of South Africa (HPCSA) and was published in March of 2023. The HPCSA is mandated by law to set minimum standards for the education and training of radiographers and clinical technologists in South Africa. This document defines the scope of practice of diagnostic radiographers in South Africa. Key elements included in this document include definitions of important terms, applications of forensic imaging, imaging modalities for forensic imaging, who can request forensic imaging, roles and responsibilities of employers, roles and responsibilities of the radiographer, health and safety (with reference to the occupational Health and Safety Act), health and welfare of radiographers, and record keeping. Regarding forensic imaging applications, this document supports the use of forensic imaging for the investigation of non-fatal injuries, locating hidden or foreign bodies, cause of death determination, and human identification. There are no specific determinations for PMCT applications.

### “Rutty’s rules”: Baseline guidance to safe postmortem computed tomography reporting [57]

Rutty’s Rules was written by Dr. Guy Rutty, a professor of forensic pathology at the University of Leicester. They were devised to guide reporting of PMCT examinations. The target audience is individuals who are non-pathology professionals who may not be aware of the pitfalls/risks involved in forensic imaging (particularly erroneous diagnoses). The ten tenants of this guide include “Teamwork, Correct training, Keeping an open mind, Information, A thorough evaluation, Interpret the PMCT images in light of the external examination findings, Expect to investigate further, The cause of death, and Specifics of PMCT reporting”. These guidelines apply broadly to the practice of PMCT. Guidelines for forensic practice in general varies depending on numerous factors including (but not limited to) country, region, jurisdiction, culture, politics, finances, and workforce. There is no “one size fits all” solution. However, working in a global scientific community necessitates the establishment of and evolution of best practice standards.

## Conclusion

From X-rays to PMCT, PMCTA, MRI and US, the variety of radiological tools in a forensic context is enormous. It is essential for the radiologist or forensic pathologist to know the advantages and limitations of all these tools in order to make better use of them. Likewise, knowledge of the recommendations written by scientific societies and published in international scientific journals is very helpful for the radiologist or forensic pathologist who will perform forensic imaging, whether in a clinical or post-mortem context. The knowledge of the correct indications in a post-mortem context is mandatory to avoid further scientific deception.

## Data Availability

Non-applicable (review).

## Code availability

Non-applicable (review).
